# Characterization of RGB-Polarization Sensor-Based Cameras

**DOI:** 10.3390/jimaging12050203

**Published:** 2026-05-07

**Authors:** Andreas Karge, Maximilian Klammer, Bernhard Eberhardt, Andreas Schilling

**Affiliations:** 1 Wilhelm Schickard Institute, Eberhard Karls University, 72076 Tübingen, Germany; 2 Faculty of Electronic Media, Stuttgart Media University, 70569 Stuttgart, Germany; 3 GMG GmbH & Co. KG, 72072 Tübingen, Germany; 4 Allied Vision Konstanz GmbH, 78467 Konstanz, Germany; maximilian.klammer@alliedvision.com

**Keywords:** imaging sensors, polarization, image formation, spectral data based camera characterization, color image reconstruction

## Abstract

This work presents a characterization method for cameras with trichromatic RGB color filter array and polarization layer (RGB-P) sensor-based imaging devices. Such sensors enable the reconstruction of color and polarization of registered scene elements, which is an important requirement in computer vision. We will present spectral responsivity measurements, which reveal different sensitivities for various color and polarization channels. Furthermore, we will discuss and model an observed chromaticity shift in registered camera signals for polarized irradiance. Both lead to inaccurate estimation of color and polarization features. In order to overcome these issues, we will present a neural-network-based model for color and polarization feature reconstruction. Essentially, it considers spectral sensitivity for polarized irradiance. Furthermore, the model takes into account that, for visualization, the color signals have to be a linear combination of polarization channels. Models were trained for selected natural and synthetic reflectance sets, as well as commonly used lighting. We evaluated the resulting performance, which yielded robust results. The method can be employed for an estimation of color and polarization features for RGB-P imaging devices. Applications can be found in photography, as well as machine and computer vision, in which object surface color rendering plays a major role.

## 1. Introduction

**Figure 1:**
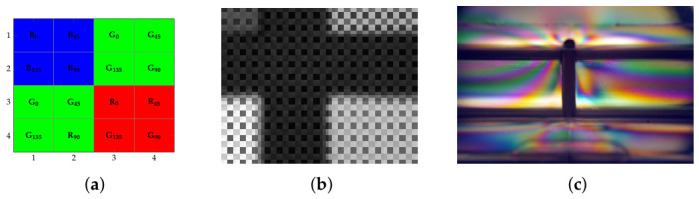
RGB-P mosaicing and image samples: (**a**) Sensor filter array structure. (**b**) Raw image sample with linear polarized irradiance. (**c**) Reconstructed color image photoelasticity sample.

Conventional imaging applications for detecting color and polarization have been well established, even using silver-halide-based analog sensors, e.g., in photoelasticity [1], as shown in Figure 1c. These types of sensors, but also digital trichromatic RGB color filter array (RGB) sensor-based cameras, require the placement of a filter in front, and they can only register one polarization direction during signal formation.

In addition to RGB sensors, RGB-P sensors have become available in recent years. An additional sensor layer contains a set of four linear polarization filters in front of the color filter layer (Figure 1a,b). Having such a registered RGB-P image that provides color and polarization features, various applications can be found in machine and computer vision, but also in photography and movie production. In machine vision, the registered features can be employed for object classification, as well as for bidirectional reflectance distribution function (BRDF) calculations. It requires color information, but also angle and degree of polarization for surface normal estimation ([2,3,4]). In the area of photographic and cinematographic applications, RGB-P images provide new artistic possibilities. Having a registered trichromatic RGB-P image, one can suppress or accentuate certain polarization directions during image post-processing for visualization. This can be done for a single image and for a movie sequence. A selection of image and image sequence samples for such applications can be found in [5]. Ensuring color constancy between images is crucial when presenting a sequence of images, such as in cinematography, where each pixel emphasizes different polarization directions.

**Figure 2:**
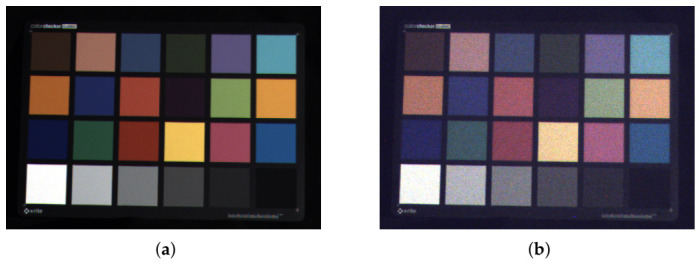
ColorChecker test chart images for different linear polarized irradiances, both normalized to white patch: (**a**) Parallel to sensor polarization direction. (**b**) Nearly perpendicular to sensor polarization direction.

All of these applications require camera characterization to reconstruct registered object color or polarization features. Errors of RGB-P sensors are discussed in [6]. Specificallly for polarized sensor irradiance, it is necessary to model the non-ideal transmittance and dispersion of this layer, which leads to chromaticity changes (Figure 2). These changes are based on the extent to which the polarizer attenuates. While Trushkina [7] discusses the physics and colorimetry of RGB imaging sensors with polarized irradiance, such models are not available for RGB-P sensors. Haila [8] investigates color accuracy with polarized irradiance, but it also only considers RGB devices.

This work is primarily driven by two key problems of RGB-P camera systems. These problems concern the signal formation and reconstruction of color and polarization features of scene objects in aforementioned applications. To address these challenges, one has to measure the spectral response of such RGB-P devices. And furthermore, a model for chromaticity and polarization feature reconstruction, particularly under different scene illuminations, is required.

Having spectral responsivites for imaging devices is a requirement in machine vision [9] as well in photography and movie production [10]. However, recent work does not take the polarization layer characteristics into account. Based on our prior work [11], we will present an extended measurement methodology to obtain RGB-P camera responses for unpolarized and polarized irradiance. Spectral responsivity can be obtained with minimal economical impact regarding measurement setup, time and cost of equipment. By employing these spectral responsivities, we will investigate spectral data-based models for color and polarization feature reconstruction. This addresses the problem that polarization features are error-prone due to the dispersion of the polarization layer under certain sensor irradiance conditions.

In the following, first, the theoretical background for RGB-P camera image formation, and color and polarization reconstruction will be discussed. Then the camera spectral sensitivity for unpolarized irradiance and chromaticity drift measurements under polarized irradiance will be presented. Based on these measurements, an estimation of spectral sensitivity for polarized irradiance will be shown. Finally, we will apply these obtained parameters to camera characterization models for different standard illuminant simulators and object reflectances, and show the evaluation results regarding chromaticity and polarization feature reconstruction.

## 2. Background

### 2.1. Scene Conditions and Signal Formation

For the signal formation model, one can consider the radiometric properties of the scene. To obtain a practical representation, the continuous spectrum of irradiance is discretized into n wavelength samples $\lambda_{i}$. The registered camera signal for a pixel is then given by

(1) $$S_{Cam,p,c}∼\sum_{i=1}^{n}\Phi \left(\lambda_{i}\right)\rho \left(\lambda_{i}\right)\tau_{c}\left(\lambda_{i}\right)\tau_{p}\left(\lambda_{i}\right)s\left(\lambda_{i}\right).$$

The spectral power distribution (SPD) of illumination Φ and spectral reflectance of object surface elements ρ describe the scene. p and c denote the polarization and color channel for the pixel element. The spectral transmittances of the color filter $\tau_{c}$ and polarization layer $\tau_{p}$ and the spectral sensitivity *s* of the semiconductor layer characterize the sensor. λ denotes the wavelength. The product ϕ(λ)ρ(λ) yields the spectral irradiance distribution Ψ.

(2) Ψ(λ)=Φ(λ)ρ(λ).

The whole sensor pixel spectral response function is

(3) $$\sigma_{p,c}\left(\lambda \right)=\tau_{p}\left(\lambda \right)\tau_{c}\left(\lambda \right)s\left(\lambda \right).$$

Therefore one can rewrite the right part of Equation (1) as $\sum_{i=1}^{n}\Psi \left(\lambda_{i}\right)\sigma_{p,c}\left(\lambda_{i}\right)$.

The object surface reflects the irradiance, along with its excitation spectrum and SPD, respectively. For polarized scene conditions, the orientation of the reflected electromagnetic waves must be also considered. For one wave with fixed wavelength λ having orientation index *j*, the irradiance is proportional to the time average squared intensity of the electric field strength $\vec{E}$.

(4) $$\Psi_{\lambda ,j}∼{\bar{{\vec{E}}_{\lambda ,j}^{2}\left(\omega \right)}}^{\;t}∼{\hat{E}}_{\lambda ,j}^{2}.$$

From Maxwell’s equations [12], this is an approximation for an electromagnetic wave with $|\vec{E}|∼|\vec{B}|$ ($\vec{B}$ magnetic field). With phase ω, $\hat{E}$ denotes the peak amplitude. Looking at light as an electromagnetic field rather than as a photon helps us later to investigate the polarization effects in detail for a RGB-P imaging sensor. The spectral irradiance distribution with discretized wavelengths $\lambda_{1}..\lambda_{n}$ for an orientation *j* is

(5) $$\begin{array}{cc} \Psi_{\lambda ,j}\; & ={\left(\begin{array}{c} \Psi_{\lambda_{1}}\\ \vdots \\ \Psi_{\lambda_{n}} \end{array}\right)}_{j}. \end{array}$$

Then, $\Psi_{j}=\sum_{i=1}^{n}\Psi_{\lambda_{i},j}$ takes into account the SPDs for all wavefronts for orientation j. The irradiance during exposure becomes

(6) $$\Psi =\left(\begin{array}{ccc} \Psi_{\lambda_{1},j_{1}} & \ldots & \Psi_{\lambda_{1},j_{m}}\\ \vdots & \ldots & \vdots \\ \Psi_{\lambda_{n},j_{1}} & \ldots & \Psi_{\lambda_{n},j_{m}} \end{array}\right)=\left(\begin{array}{ccc} \Psi_{\lambda j_{1}} & \ldots & \Psi_{\lambda j_{m}} \end{array}\right).$$

During exposure, the sensor pixel registers

(7) $$\Psi =\sum_{j=1}^{m}\sum_{i=1}^{n}\Psi_{j}\left(\lambda_{i}\right)\sigma \left(\lambda_{i}\right).$$

**Figure 3:**
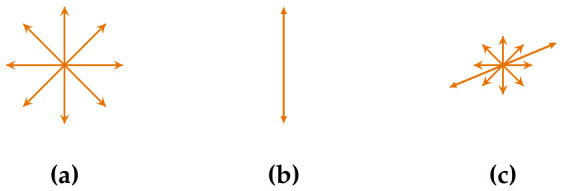
Selected intensity distribution examples of sensor irradiances regarding different scene conditions: (**a**) Unpolarized (**b**); full polarized; (**c**) superposition of **a** and **b**.

When dealing with polarized irradiance, the SPD is weighted differently depending on the orientations j. Figure 3 shows some possible scenarios for incident light during exposure time. First, **a** shows the typical unpolarized case, while **b** shows fully linear polarized irradiance of a specular highlight. A superposition of a special case of specular and a Lambertian reflectance is shown in **c**. Circular polarization states can also appear, but are not investigated. For incoherent irradiance, the distribution can be decomposed into two components perpendicular to each other. But for later use in camera characterization input data setup (see Section 5.2), we consider another decomposition method. If we replace j by using the unity vector ${\vec{e}}_{j}$ denoting orientation of ${\vec{E}}_{j}$, we can then define an amplitude distribution function $d_{\epsilon }\left({\vec{e}}_{j}\right)$. Furthermore, we normalize it so that for orientation without attenuation, $d_{\epsilon }\left({\vec{e}}_{max}\right)=1$, having the maximum SPD $\hat{\Psi }=\Psi_{{\vec{e}}_{max}}$. One can then write the following.

(8) $$\begin{array}{c} \Psi \left(d_{\epsilon }\right)=\left(\begin{array}{ccc} d_{\epsilon }\left({\vec{e}}_{1}\right){\hat{\Psi }}_{\lambda } & \ldots & d_{\epsilon }\left({\vec{e}}_{m}\right){\hat{\Psi }}_{\lambda } \end{array}\right). \end{array}$$

For the unpolarized state, it is $d_{\epsilon }=const.=1$, and for completely linear polarized $d_{\epsilon }\left({\vec{e}}_{max}\right)=1$, but 0 otherwise. Irradiance for signal formation becomes

(9) $$\Psi =\sum_{j=1}^{m}\sum_{i=1}^{n}d_{\epsilon }\left({\vec{e}}_{j}\right)\hat{\Psi }\left(\lambda_{i}\right)\sigma \left(\lambda_{i}\right).$$

### 2.2. Polarization Layer Characterization

The sensor irradiance $\Psi (d_{\epsilon })$ is passing the polarization layer, and the color filter layer, and finally, it gets registered by the photosensitive layer. In the following, the terminology “analyzer” will be used for the polarization layer because it analyzes the state of incoming polarization. An ideal analyzer would fully absorb the perpendicular component of the incident wave $\vec{E}$, while allowing the parallel component to pass through. It becomes

(10) $$\begin{array}{c} {\vec{e}}_{a}{\vec{E}}_{\lambda ,j}=\cos\left(\psi_{A}\right){\hat{E}}_{\lambda ,j}, \end{array}$$

**Figure 4:**
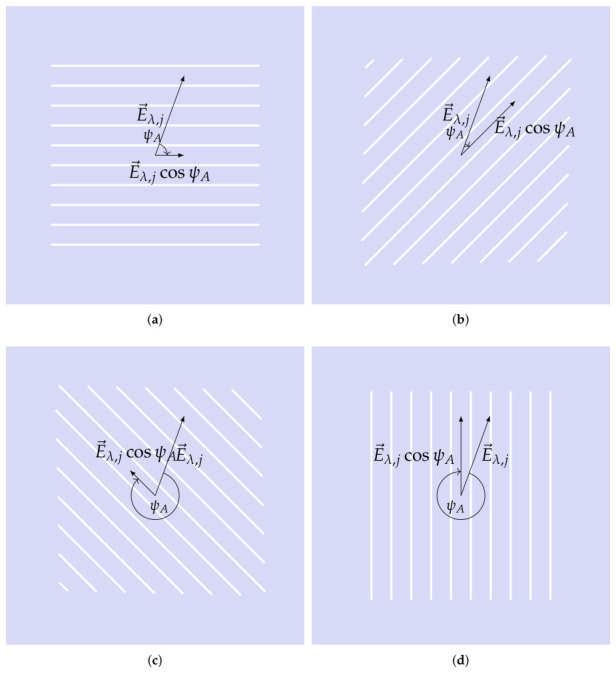
Incident electric field and its effective component for the polarization sensor array: (**a**) 0°; (**b**) 45°; (**c**) 135°; (**d**) 90°.

${\vec{e}}_{a}$ denotes the orientation of the analyzer. $\psi_{A}$ is the angle between the analyzer and incoming electric field strength orientation (Figure 4). The irradiance for color filter layer after passing through the analyzer becomes

(11) $$\Psi_{a,\lambda ,j}∼\cos^{2}\left(\psi_{A}\right){\hat{E}}_{\lambda ,j}^{2},$$

It follows $\cos^{2}$, known as the MALUS law [13]. The overall irradiance after passing the analyzer results is

(12) $$\begin{array}{c} \Psi_{a}\left(d\left(\epsilon \right)\right)=\left(\begin{array}{ccc} \cos^{2}\left(\psi_{1}\right)d_{\epsilon }\left({\vec{e}}_{1}\right){\hat{\Psi }}_{\lambda } & \ldots & \cos^{2}\left(\psi_{k}\right)d_{\epsilon }\left({\vec{e}}_{m}\right){\hat{\Psi }}_{\lambda } \end{array}\right). \end{array}$$

This irradiance passes the color filter with the spectral transmittance $\tau_{C}\left(\lambda \right)$ and hits the sensitivity layer with spectral sensitivity s(λ). The RGB-P pixel response function for a certain orientation, color channel *c* and analyzer *A* is

(13) $$\begin{array}{c} \sigma_{p,c}\left(\lambda \right)=\cos^{2}\left(\psi_{A}\right)\tau_{a}\left(\lambda \right)\tau_{c}\left(\lambda \right)s\left(\lambda \right). \end{array}$$

Irradiances from wavelets with different orientation hit the polarization layer of the RGB-P pixel during exposure time Δt. For equal distributed orientations during exposure time, the resulting signal becomes

(14) $$\begin{array}{cc} S_{Cam,p,c} & \approx \sum_{k=0}^{l-1}\langle d_{\epsilon }\left(k\Delta \phi \right){\hat{\Psi }}_{\lambda },\cos^{2}\left(k\Delta \phi -\alpha_{A}\right)\sigma_{p,c,\lambda }\rangle , \end{array}$$

where

- Δϕ=2π/l denotes the angle sample steps with l as number of samples for incident wave orientations.
- kΔϕ denotes the orientation of the incident wave in sensor plane, and $k\Delta \phi -\alpha_{A}$ the projection angle with analyzer orientation $\alpha_{A}$.
- $d_{\epsilon }\left(k\Delta \phi \right)$ denotes the amplitude for orientation angle.
- p = (0,45,90,135) denotes the polarization layer angle α in degrees, and c = (R,G,B) the color channel.
- $\sigma_{p,c,\lambda }$ denotes the sensitivity sample vector ${\left(\sigma_{p,c}\left(\lambda_{1}\right)\ldots \sigma_{p,c}\left(\lambda_{n}\right)\right)}^{T}$.

Previous considerations are only valid for an ideal polarizer. Real polarizers deviate from it, i.e., the MALUS law is not fulfilled. This behavior can be described by the spectral extinction ratio as a result of dispersion and other optical properties, e.g., discussed for polymer-based polarisers in [14]. For example, an orthogonally positioned analyzer will not completely absorb a fully polarized set of waves, and it is

(15) $$\frac{\Psi_{a}\left(\psi_{A}=\frac{\pi }{2}\right)}{\Psi_{a}\left(\psi_{A}=0\right)}\neq 0=cos^{2}\left(\psi_{A}=\frac{\pi }{2}\right).$$

For our observed chromaticity drift as previously depicted in Figure 2, it is due to varying attenuation depending on wavelength. This can be written as

(16) $$\frac{\Psi_{a,\lambda_{1}}\left(\psi_{A}\right)}{\Psi_{a,\lambda_{1}}\left(\psi_{A}=0\right)}\neq \frac{\Psi_{a,\lambda_{2}}\left(\psi_{A}\right)}{\Psi_{a,\lambda_{2}}\left(\psi_{A}=0\right)}.$$

The spectral response σ is material-dependent for the polarization layer with $\tau_{a}$. It can be modeled as a function of the angle:

(17) $${\tilde{\sigma }}_{p,c}\left(\lambda ,\psi_{A}\right)=\tau_{a}\left(\lambda ,\psi_{A}\right)\tau_{C}\left(\lambda \right)s\left(\lambda \right).$$

For RGB-P sensors created by a microlithographic process, e.g., discussed in [15], the dispersion is not specified by the manufacturer. $\tilde{\sigma }\left(\lambda ,\psi_{A}\right)$ is not known but can be approximated as investigated later in Section 4. An RGB-P camera characterization therefore must be aware of those characteristics. The registered signal for the whole polarized irradiance becomes

(18) $$S_{Cam,p,c}∼\sum_{k=0}^{n-1}\langle d_{\epsilon }\left(k\Delta \phi \right){\hat{\Psi }}_{\lambda },{\tilde{\sigma }}_{p,c,\lambda }\left(k\Delta \phi -\alpha_{A}\right)\rangle .$$

We consider the signal formation as a linear combination. It is formed by the orthogonal and parallel part of incident wave, regarding the orientation to the analyzer. Let us denote sensitivity for the parallel component as $\sigma_{\|}$, and for the perpendicular component as $\sigma_{⊥}$. Then, the registered signal becomes

(19) $$S_{Cam,p,c}=\sum_{k=0}^{n-1}\left(\langle d_{\epsilon }\left(k\Delta \phi \right){\hat{\Psi }}_{\lambda },cos^{2}\left(k\Delta \phi -\alpha_{A}\right)\sigma_{\|}+sin^{2}\left(k\Delta \phi -\alpha_{A}\right)\sigma_{⊥}\rangle \right).$$

### 2.3. Scene Feature Reconstruction

First, we consider color reconstruction. In general, RGB camera sensors do not fulfil the LUTHER condition [16]. To put it another way, they are not colorimetric. The sensor signals are not a linear combination of the human standard observer tristimulus $C_{CIE}$ as defined by the Commission Internationale de l’Éclairage (CIE), because the channels’ spectral responsivities are different from those of the human standard observer. For a single signal, formation follows Equation (1), which treats lighting as a standard illuminant simulator SPD. For a human standard observer with observer response $s_{obs}=\left(\bar{x},\bar{y},\bar{z}\right)T$, the tristimulus is

(20) $$C_{CIE}∼\sum_{i=1}^{n}\Phi_{std}\left(\lambda_{i}\right)\rho \left(\lambda_{i}\right)s_{obs}\left(\lambda_{i}\right).$$

$\Phi_{std}\left(\lambda \right)$ denotes a standard illuminant, while the already introduced Φ(λ) of real lighting denotes a standard illuminant simulator. Irradiance for a standard illuminant becomes $\Psi_{std}=\Phi_{std}\left(\lambda_{i}\right)\rho \left(\lambda_{i}\right)$. RGB camera characterization is defined as follows:

(21) $$C_{CIE}=\left(\begin{array}{c} X\\ Y\\ Z \end{array}\right)=M_{C}\left(\begin{array}{c} R\\ G\\ B \end{array}\right)=M_{C}S_{Cam}.$$

The model $M_{C}$ transforms the non-colorimetric signals into a standardized human observer tristimulus with virtual primaries X,Y, and *Z*. $M_{C}\in R^{3\times 3}$ is a commonly used model, but one can also choose higher-dimensional approaches [17,18,19]. Generally, we can estimate the model parameters by solving the minimization problem.

(22) $$\min_{M_{C}}\frac{1}{2}{\left\|C_{CIE}-M_{C}S\right\|}_{2}^{2}.$$

S denotes the set of signals for training data, and C represents the corresponding tristimuli. Optionally, the model parameter set can be reduced by using the white-point preserving approach [20]. The CIE-XYZ domain does not have an equal distant perception metric. E.g., for CIE-Lab 1976, where the distance metric is equally perception-based, the problem to be solved becomes

(23) $$\min_{M_{C}}\frac{1}{2}{\left\|f\left(C_{CIE}\right)-f\left(M_{C}S\right)\right\|}_{2}^{2},$$

where *f* is a nonlinear function converting the tristimulus into CIE-Lab values. Then, the model can only be approximated by numerical solvers. E.g., the AMPAS-ACES standard uses this approach [10]. To achieve lightness independence, one can reduce the problem to preserve only the chromaticities. By calculating the camera chromaticities from the measured signals with

(24) $$s=\left(\begin{array}{c} r\\ g\\ b \end{array}\right)=\frac{1}{S_{R}+S_{G}+S_{B}}\left(\begin{array}{c} S_{R}\\ S_{G}\\ S_{B} \end{array}\right),$$

and the chromaticity of a human standard observer from the tristimulus

(25) $$c_{CIE}={\left(\begin{array}{c} x\\ y\\ z \end{array}\right)}_{CIE}=\frac{1}{X+Y+Z}{\left(\begin{array}{c} X\\ Y\\ Z \end{array}\right)}_{CIE}.$$

the model becomes

(26) $$M_{c}:{\left(r,g\right)}_{Cam}\mapsto {\left(x,y\right)}_{CIE}.$$

Methods are discussed in [21], and [22,23,24], the latter one using hue plane preserving camera characterization.

Besides color reconstruction, for RGB-P cameras, we also consider the polarization features of the scene. For incoherent irradiance of an RGB-P sensor, the polarization state can be described by the STOKES vector

(27) $$S_{c}=\left(\begin{array}{c} S_{0,c}\\ S_{1,c}\\ S_{2,c} \end{array}\right)=\left(\begin{array}{c} \frac{1}{2}\left(S_{0,c}+S_{45,c}+S_{90,c}+S_{135,c}\right)\\ S_{0,c}-S_{90,c}\\ S_{45,c}-S_{135,c} \end{array}\right),c=\left(R,G,B\right),$$

as long as the fourth component $S_{3,c}$ is neglected. I.e., it is assumed that circular polarized light is not present but only unpolarized, partially, and linear polarized. The degree of polarization (DoP) is

(28) $$DoP_{c}=\frac{\sqrt{S_{1,c}^{2}+S_{2,c}^{2}}}{S_{0,c}},$$

and angle of polarization (AoP) given by

(29) $$AoP_{c}=\frac{1}{2}\arctan\frac{S_{2,c}}{S_{1,c}}.$$

$DoP_{c}\left(\lambda \right)$ and $AoP_{c}\left(\lambda \right)$ are assumed to be constant over the wavelength for each color channel. We combine both polarization features into a polarization feature set.

(30) $$S_{c}={\left(AoP_{c},DoP_{c}\right)}^{T}.$$

These equations are valid only for an ideal polarizer. As discussed in Section 2.1, the registered signals of an RGB-P sensor are a function of the sensitivity $\sigma {\left(\lambda ,\psi \right)}_{A,C}$. The measured Stokes components $\tilde{S}$ are therefore afflicted with systematic errors. The resulting error-prone polarization feature is ${\tilde{S}}_{C}$. Defining ${\tilde{S}}_{RGB}={\left({\tilde{S}}_{R},{\tilde{S}}_{G},{\tilde{S}}_{B}\right)}^{T}$, one must consider a model for approximation of polarization features.

(31) $$M_{P}:{\tilde{S}}_{RGB}\to S.$$

### 2.4. Visualization of RGB-P Images

By applying camera characterization and display calibration, we represent RGB images on RGB displays, achieving standardized color tristimuli. For RGB-P images, every color channel provides four polarization channels. Since human observers cannot distinguish different polarization states (except the HAIDINGER’s brush phenomenon [25]), only RGB displays exist. For visualization, a linear combination of all polarization channels will be used. To obtain the final combined signal $S_{\Sigma }$, define a vector $w={\left(w_{0},w_{45},w_{90},w_{135}\right)}^{T}$ of weighting coefficients for polarization channels and apply it to a matrix $S=(S_{0},S_{45},S_{90},S_{135})$.

(32) $$S_{\Sigma }=Sw.$$

As discussed above, to have lightness independence, one can reduce the weighted signal to the chromaticity domain. The chromaticity of the combined signal (all signals ≥ 0) becomes

(33) $$\begin{array}{cc} s_{\Sigma } & =\frac{1}{\left|\right|S_{\Sigma }{\left|\right|}_{1}}S_{\Sigma }. \end{array}$$

To ensure constant CIE chromaticities, a model as follows must be obtained:

(34) $$M_{\Sigma }:s_{\Sigma }\to c_{CIE}.$$

### 2.5. Problem and Generalized Model

Characterizing an RGB camera requires a constant response that is independent of the polarization channel, and even independent from the polarization of irradiance itself. For an RGB-P sensor, the image formation is much more complex. Each signal is influenced by the polarization direction of light on the sensor’s surface, the angle between the polarization and pixel analyzer orientation, and the selected projection angle. Moreover, ensuring color constancy is crucial for visualizing RGB values for time variable projection angles in image sequences. Section 2.3 discussed conventional mathematical models that are not applicable. They suffer from observed chromaticity drifts. In summary, a raw RGB-P image serves two distinct purposes: the application of polarization and color features for classification applications and the visual presentation. For both purposes, the following tasks must be considered:

- Classification: Signal to standard chromaticities mapping, degree and angle of polarization reconstruction.
- Visual presentation of an RGB-P image: Combined signal to standard color domain mapping, ensuring color constancy for different linear combinations of signals.

Now a generalized model is defined, which combines $M_{\Sigma }$ (Equation (34)) and $M_{P}$ (Equation (31)) to approximate color and polarization features.

(35) $$M_{Pc}\left(M_{P},M_{\Sigma }\right):\left(\begin{array}{c} r_{\Sigma }\\ g_{\Sigma }\\ b_{\Sigma }\\ {\tilde{AoP}}_{R}\\ {\tilde{DoP}}_{R}\\ {\tilde{AoP}}_{G}\\ {\tilde{DoP}}_{G}\\ {\tilde{AoP}}_{B}\\ {\tilde{DoP}}_{B} \end{array}\right)=\left(\begin{array}{c} s_{\Sigma }\\ {\tilde{S}}_{R}\\ {\tilde{S}}_{G}\\ {\tilde{S}}_{B} \end{array}\right)\mapsto \left(\begin{array}{c} c_{CIE}\\ S \end{array}\right)=\left(\begin{array}{c} x_{CIE}\\ y_{CIE}\\ AoP\\ DoP \end{array}\right).$$

In this model, all chromaticities r, g, and *b* are used. In the ideal case, having r+g+b=1, this is overdetermined, but since we have signal and numerical noise, this term was later on applied during camera characterization.

Based on the RGB-P camera image formation we discussed in this chapter, for a characterization model based on Equation (19), the two spectral responsivities $\sigma_{\|}$ and $\sigma_{⊥}$ must be obtained. The next chapter describes the chosen experimental setup and measurement methods in order to obtain this camera responses.

## 3. Performed Measurements

### 3.1. Spectral Response Measurements for Unpolarized Irradiance

**Figure 5:**
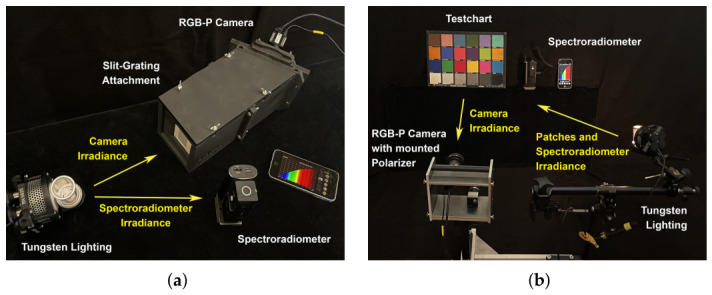
Experimental setup: (**a**) Spectral responsivity measurement setup for unpolarized irradiance. (**b**) Test chart measurement setup for responsivity estimation under polarized irradiance.

An overview of methods for camera sensitivities estimations is given in [26]. We employ the methodology and setup of the Open Film Tools camera response measurement method [11] (OFTEx). Figure 5a shows our principal measurement setup for the OFTEx method.

All measurements were taken with an IDS machine vision camera GV-508x CP-Q (GV-5080CP-Q-GL R2, Serial Number 4103534131) from IDS Imaging Development Systems GmbH, Obersulm, Germany and a Pentax 1:1.4/16 lens (Ricoh Imaging Company, Ltd., Tokyo, Japan). The exposure time was in a range of 500 ms up to 2 s. During the measurement, we aligned the camera horizontally. Measurements carried out beforehand with rotated sensor positions showed no deviations in registered signals. We conducted this to assess the potential impact of sensor analyzer orientations. The spectroradiometer UPRTEK MK350D was used for all spectral measurements.

**Figure 6:**
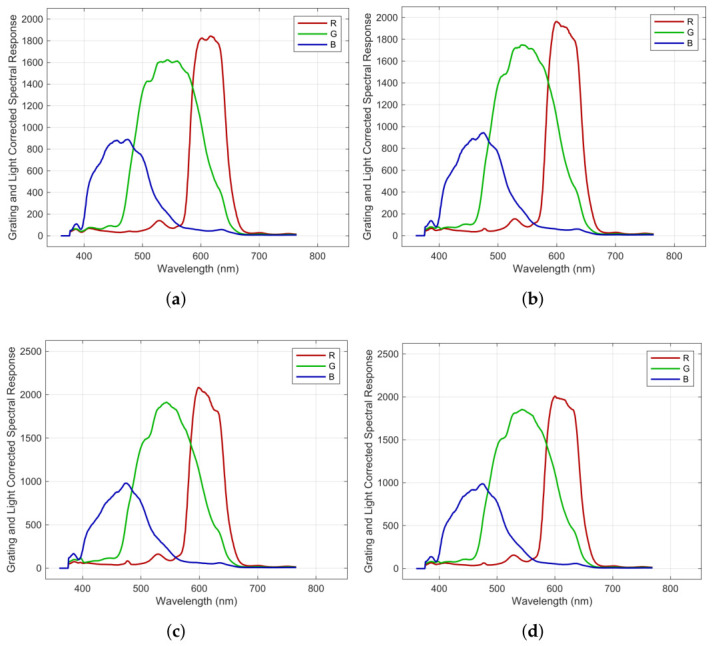
OFTEx [11] corrected registered code values of spectal response for all 4 polarization angles for unpolarized irradiance: (**a**) 0° (**b**) 45° (**c**) 90° (**d**) 135°.

Figure 6 represents our measurement results. In detail, it shows all four polarization layers measured RGB responses for unpolarized irradiance separately. Responsivity data can be found in [27] (Folder “01CameraMeasurements”). The measured sensitivities are different. The basic assumption that all four polarization layers have the same responses for a color channel is false. It must be rewritten as a function of color *C* and polarizer *A* channel denoted as $\sigma_{C,A}$. For color signal normalization, one might estimate and apply the scaling vector for intensity adjustment. It can be obtained from part 3 of the OFTEx, which captures a chart, e.g., from white patch samples.

With this measurement setup, we now obtained the overall sensitivity $\tilde{\sigma }$ regarding Equation (17). But our discussed image formation model requires us to split the sensitivity into parallel and perpendicular parts (Equation (19)). We will measure chromaticities under polarized irradiance as shown in the following. Out of these measurements, we will estimate $\sigma_{⊥}$ and $\sigma_{\|}$.

### 3.2. Measurements for Polarized Irradiance

For creation of polarized irradiance conditions, we used an adapted experimental setup method from the OFTEx, as seen in part 3. We applied an additional linear polarization filter in front of the camera to create linear polarized irradiance on the sensor’s surface. This is known as a polarizer-analyzer setup in optics. It generates polarized light from the first polarization filter element, while the second polarization filter from trichromatic RGB-P layers analyses the orientation for specific filter orientations. If the filter orientations are the same, the extrema represent maximal transmission, and if they are oriented orthogonally, the opposite represents maximal attenuation. We must consider the spectral transmittance of the polarizer $\tau_{\lambda }$ for this setup. In this regard, the registered signal must be normalized. We measured $\tau_{\lambda }$ using the same setup as before, but we replaced the camera with a spectroradiometer. Beforehand, we conducted a series of measurements with various polarizer and spectroradiometer orientations, demonstrating the independence of measurements from the alignment. It must be noted, that, in general, the polarizer will also deviate from the ideal polarization as discussed above. It can be characterized using the same method as we use for sensor characterization, but we will neglect this error in the following.

The measurement setup uses a 45°/0° geometry. It involves a TUNGSTEN light source at a 45° angle relative to the test chart surface. The camera, on the other hand, is positioned at 0° from the test chart surface, viewing the chart directly from above. The distances are for both, camera and lighting, 1.5 m regarding the test chart center. Figure 5b shows the principal measurement setup for the test chart measurement.

Nine different orientation angles of the polarizer in front of the camera system were applied. The orientation angle was increased by steps of 22.5 degrees. We started by measuring the maximum green signal for polarization channel 0 in $\frac{\pi }{8}$ and gradually increased it to the maximum transmittance for the opposite orientation at π, $\frac{\pi }{2}$ in between, therefore having approximately maximum absorption. For the other sensor polarization channels, the change in transmittance is shifted by $\frac{\pi }{4}$. As before, the ColorChecker (CC) and additionally ColorChecker Digital SG (CC-DSG) charts were used. The latter chart incorporates the previous one at the center, along with additional surrounding patches such as for skin tones or semigloss reflectance.

**Figure 7:**
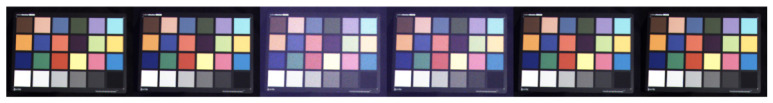
White patch normalized and averaged sample images for different polarizer orientations and polarization layer $P_{0}$.

Figure 7 shows measured ColorChecker angle samples 3–8 (45–157.5°) of $P_{0}$, normalized to white patch. The measured chromaticities near full extinction can be employed to estimate the spectral response $\sigma_{⊥}$.

## 4. Spectral Response for Polarized Irradiance

### 4.1. Response Estimation

For spectral response estimation under polarized irradiance, one has to model the dispersion of wire-grid polarizing elements in the polarization layer of the RGB-P sensor. In our prior work [11], we investigated dispersion of echelette gratings in a slit-grating setup. We demonstrated that a second-order polynomial is sufficient for certain gratings fabricated using a micro-gravure process. However, in this work, we have to model unknown wire-grid polarizers fabricated using a microlithographic process. We setup γ(λ) as a general polynomial based model for dispersion. We approximate the response $\sigma_{⊥}$ by using the sensitivity under unpolarized irradiance $\tilde{\sigma }$ and γ(λ) having $\sigma_{⊥c,p}\left(\lambda \right)\approx \gamma \left(\lambda \right){\tilde{\sigma }}_{c,p}\left(\lambda \right)$. The signal for perpendicular irradiance becomes

(36) $$S_{⊥Cam,c,p}∼\langle \tau^{-1}\left(\lambda \right)\Psi \left(\lambda \right),diag\left(\gamma \left(\lambda \right)\right){\tilde{\sigma }}_{c,p}\left(\lambda \right)\rangle .$$

**Figure 8:**
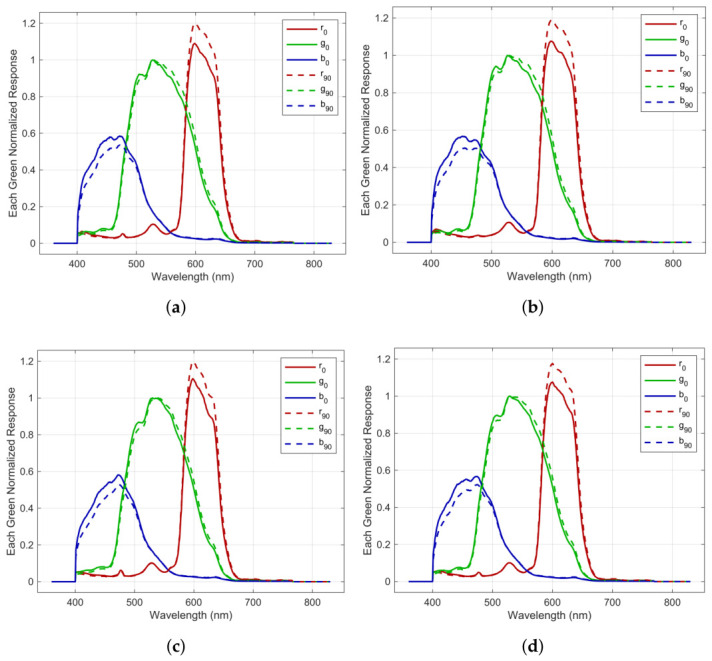
Green normalized responsivities for parallel ($r_{0},g_{0},b_{0}$) and orthogonal ($r_{90},g_{90},b_{90}$) irradiance for all 4 sensor polarizer channels: (**a**) 0°; (**b**) 45°; (**c**) 90°; (**d**) 135°.

**Figure 9:**
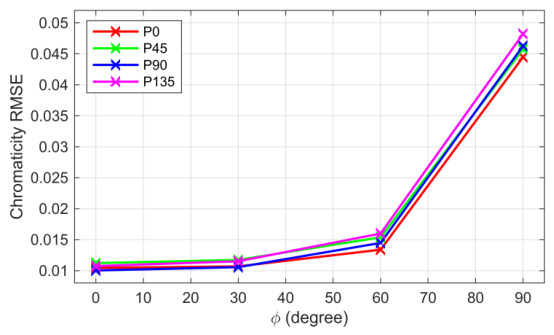
Chromaticity RMSE for all polarization channels over projection angle, the measured sample points are marked with x.

Term $\tau^{-1}\left(\lambda \right)$ compensates the front-mounted polarization filter transmittance. The minimization problem can then be formulated as

(37) $$\min_{\Gamma }\frac{1}{2}{∥{\tilde{S}}_{⊥Cam,p}-S_{⊥Cam,p}\left(\gamma \right)∥}_{2}^{2},$$

whereas Γ is the coefficient vector of γ(λ) and ${\tilde{S}}_{⊥Cam,p}$ are the set of registered RGB signals for perpendicular irradiance. And we constrained the solution allowing only estimated signals ≥0 and $\sigma_{⊥}\left(\lambda \right)\geq 0$ respectively. Initial guesses for sensitivity were obtained by scaling sensitivity for unpolarized irradiance down according to the ideal $\cos^{2}\left(\psi \right)$ rule. By solving this problem, we obtain $\sigma_{⊥}$ and since $\sigma_{⊥}\ll \tilde{\sigma }$, we choose $\sigma_{\|}\approx \tilde{\sigma }$. In our case, we proved that a first-order linear polynomial was sufficient. Figure 8 shows the estimated responses for all polarization and color channels regarding parallel and orthogonal linear polarized sensor irradiance. The chromaticities’ root mean square error between measured patches and estimations by applying polarized and unpolarized responses in Equation (19) are shown in Figure 9. Responsivities are available in [27] (Folder “02EstimatedSpectralResponsivitiesForPolarisedConditions”).

### 4.2. Chromaticity Drift

**Figure 10:**
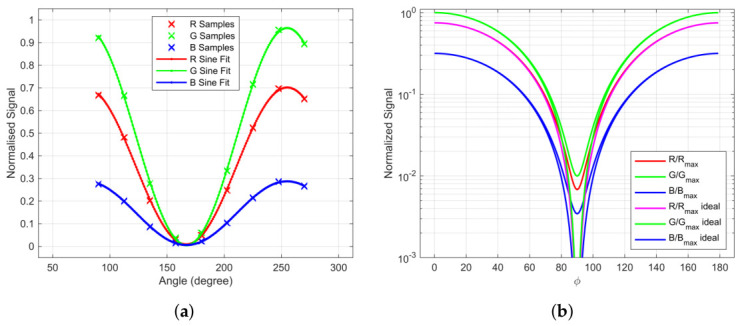
Signals over polarization angle for ColorChecker white patch and polarization channel $P_{0}$: (**a**) Measured and normalized signals. (**b**) Estimated and normalized signals using Equation (36) by employing obtained $\sigma_{\|}$ and $\sigma_{⊥}$ over polarization angle for ColorChecker white patch and polarization channel $P_{0}$ compared to ideal MALUS law.

As illustrated in Figure 7 the measurements under polarized conditions clearly show the chromaticity drift, e.g., in the case of ColorChecker white patch signals for different polarization angles as shown in Figure 10a. The data is based on captured test chart images available in [27] (Folder “01CameraMeasurements”). Figure 10b shows the normalized signals calculated with Equation (36) applying $\sigma_{\|}$ and $\sigma_{⊥}$.

**Figure 11:**
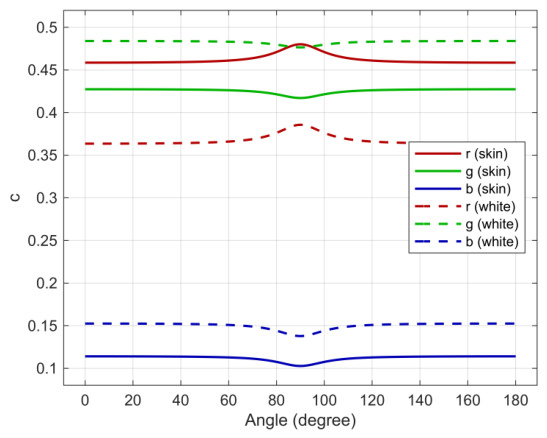
White and caucasian skin tone chromaticities over polarization angle (*P_0_*).

**Figure 12:**
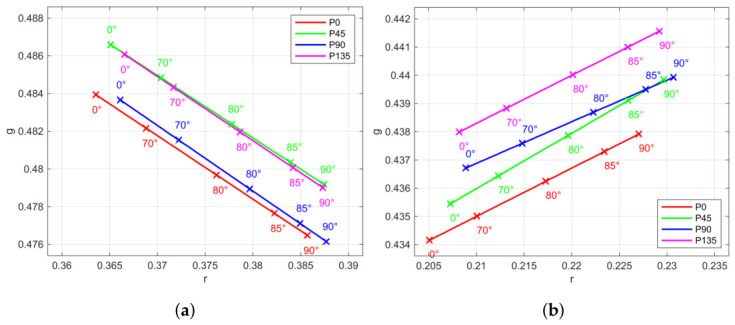
Chromaticity changes regarding angle between incident linear polarized light and analyzer orientation (ψ) for all four sensor polarization channels and selected patches: (**a**) White. (**b**) Blue.

**Figure 13:**
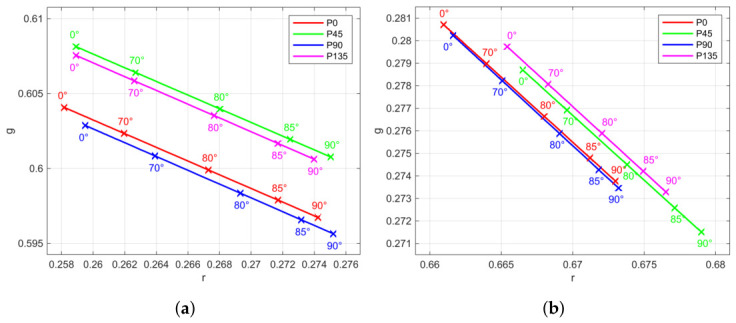
Chromaticity changes regarding angle between incident linear polarized light and analyzer orientation (ψ) for all four sensor polarization channels and selected patches: (**a**) Green. (**b**) Red.

Variation in hue and saturation can be observed. For one polarization channel and selected patches, they are shown in Figure 11. One cause is the spectral attenuation of the polarization layer [28]. In particular, we observed different oriented chromaticity changes for certain patches under linear polarized irradiance conditions. Each polarization channel behaves uniquely (Figure 12 and Figure 13), and the spectral absorption of the polarization layer depends on polarization orientation. On the other hand, the measurements revealed no phase alignment errors between the channels with different polarization angles.

### 4.3. Proposed Extension of OFTEx Responsivity Measurement Method for RGB-P Devices

**Figure 14:**
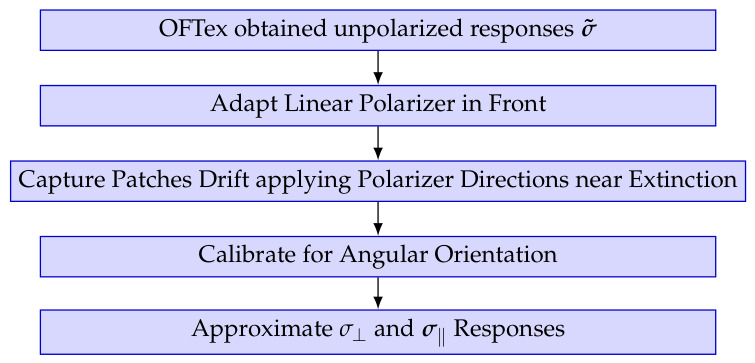
OFTEx Algorithm extension for RGB-P camera characterization.

The previously described polarizer-analyzer setup and responsivity estimation for polarized irradiance can extend the OFTEx measurement method [11] to facilitate an RGB-P camera characterization. Figure 14 shows the required additional steps.

With the obtained responsivities, and different illumination and object reflectance sets, a data setup for camera characterization can be done, which we will now discuss in detail.

## 5. Data Setup for RGB-P Camera Characterization

In Equation (35), the input feature set refers to scene conditions, RGB-P sensor characteristics, and output rendering. The scene conditions are lighting, object reflectance, and polarization state. The sensor is characterized with irradiance polarization state-dependent sensitivities. The camera chromaticities also refer to the output rendering, i.e., the weighted linear combinations of signals. The lighting and reflectance data sets that were used will now be discussed.

### 5.1. Selection of Lighting and Reflectance Data Sets

We selected commonly used standard illuminant simulators from [29] (data set at [30]) for SPD Φ(λ). The selection contains samples for a light-emitting diode (LED) and hydrargyrum medium-arc iodide (HMI) as daylight, as well as Tungsten (T) and phosphorescence (P) as ambient lighting. For the latter, the black body radiation, along with its correlated color temperature (CCT), provides the output SPD, whereas for daylight, the CIE standard illuminants were chosen. Sample sets were selected as shown in Table 1.

Furthermore, we selected unpolarized object reflectance sets from movie production applications [31] (AMPAS Color Encoding System (ACES)) and from photographic industry standards [32] (ISO/TR 16066 standard (SOCS)), and the skin tone subset from ISO/TR 16066 standard (SOCS skin reflectances (SOCS-S)). We also looked at commonly used test chart-based reflectance sets, such as the ColorChecker test chart (CC) and the ColorChecker Digital SG test chart (CC-DSG). The latter also includes semigloss patches. Furthermore, we took into account synthetically generated, simulated reflectance spectra [33] (LOGVINENKO reflectances [34] (LOGV)). This defines a set of GAUSSIAN distributed reflectances with parameters for amplitude, full-width half maximum (FWHM), and peak wavelength. Since the chromaticity approach is used here, only the maximum normalized reflectance κ = 1 is chosen. For peak wavelength μ, we sample 360 to 830 nm (CIE human standard observer range) in 15 nm steps. We chose 15 nm because commonly used spectroradiometers nowadays provide a spectral resolution in that range. The FWHM parameters σ were sampled in the range of 100 nm to 3200 nm, with a step size of 50 nm. Previous tests also used unnatural narrow band reflectances with σ down to 1 nm, but this resulted in poor performance. It must be noticed that [33] can also be employed for simulation SPD of lighting [34].

### 5.2. Setup of Input

The different polarization states of object surface-reflected wavefronts $d_{\epsilon }\left(\phi \right)$ (Equation (8)) are simulated. Without loss of generality, we will consider cases where the polarization state is assumed, forming an ellipsis. Then, it becomes a function of ellipsis eccentricity ϵ. $d_{\epsilon }$ is then defined by major and minor focus distances a and b. The parametric equation for the ellipsis is

(38) $${\left(\begin{array}{c} x(\phi )\\ y(\phi ) \end{array}\right)}_{\epsilon }={\left(\begin{array}{c} a\cos\phi \\ b\sin\phi \end{array}\right)}_{\epsilon }.$$

ϕ defines the orientation angle corresponding to j and ${\vec{e}}_{j}$ respectively, and $b=a\sqrt{1-\epsilon^{2}}$. In the following example, having a=1, the ellipsis major axis is considered to be oriented vertically; then, one can write

(39) $$d_{\epsilon }\left(\phi \right)=\frac{\sqrt{1-\epsilon^{2}}}{\sqrt{1-\epsilon^{2}\cos^{2}\phi }}.$$

**Figure 15:**
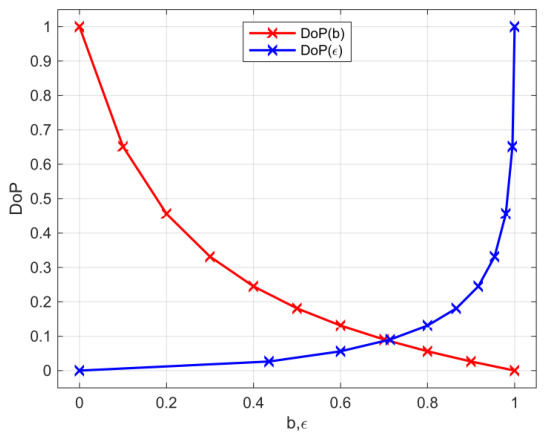
DoP as a function of irradiance distribution ellipsis parameters b, and ϵ respectively. The applied sample points are marked with x.

The DoP samples were obtained by variation of minor axis ellipsis parameter b sampled by steps 0.1 ∈[0,1]. Figure 15 shows DoP as a function of the ellipsis parameters and our selected sample points.

**Figure 16:**
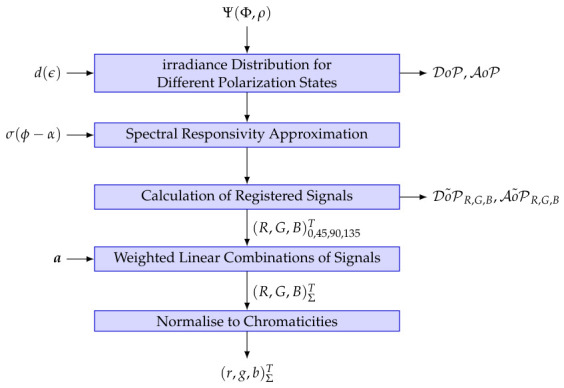
Training data input preparation.

The AoP samples were sampled by steps of one degree $\in [0^{\circ },355^{\circ }]$. For an unpolarized state, DoP becomes 0 and AoP is not defined. Since our model uses all three measured chromaticities, we ensure a random setup of AoP for that case. For every wavelet with irradiance $\Phi_{i,j}$ and its orientation ϕ=ψ−α relative to the analyzer angle, the spectral response for certain colors and polarization channel C,P is approximated previously described in Section 4. Having that, the calculation of registered signals ${\tilde{S}}_{P,C}$ can be done by integration over all wavelets for different orientations. For the weighted linear combinations, a weighting sampling rate of 0.25 ∈[0,1] (except combination (0,0,0,0)) was chosen. Figure 16 shows the whole algorithm for one sensor element.

It should be noted that this is only an ad hoc, simplified simulation of possible variations discussed in Section 2.1. In general, reflectances can also occur, where the spectral reflectance distribution varies with angle. Such a situation can arise from the superposition of a specular highlight and diffuse Lambertian reflectance, as occurs, for example, on metallic as well as on non-conductive glass or polymer surfaces. In extreme cases, the sensor irradiance may be degraded and reduced to the SPD of the illumination itself. An example is the imaging of a human face, where the subject wears glasses. This can be modeled as a superposition of Fresnel reflections at the air–glass and eye boundaries, while the underlying skin behind the glass may behave approximately as an ideal Lambertian diffuser. The object reflectance sets in [31] and also the Colorchecker Digital SG provide certain patches representing specular highlight or semigloss reflectance, which partially addresses this issue in our trained model. For such situations, one can simulate or even create ground truth data to train an application-specific model.

### 5.3. Setup of Output

In order to get output data SPD, for every input SPD, the daylight standard illuminant or PLANCK black body radiation source CCT equivalent was used. The setup of the output uses the same object reflectance sets as in the input. Irradiance then becomes $\Psi_{std}=\Phi_{std}\left(\lambda \right)\rho \left(\lambda \right)$. The observer responses are provided by the CIE two-degree standard observer. Equations (20) and (25) determine the tristimulus primaries. As discussed, the human observer is not able to detect the polarization state; the output chromaticities were set as equal for different polarization states. The expectation values for AoP and DoP are given by the simulated sample values previously calculated in the input setup.

### 5.4. Data Pre-Processing

Extrapolation is required if SPDs in the CIE wavelength range of 360 to 840 nm are not available. If resolution is not 1 nm, an interpolation must be applied. For instance, Wang [35] and the CIE standard committee [36] have discussed various methods. Here, the latter one was applied. Extrapolated values were set to zero and all samples were resampled to 1 nm steps. All data is already normalized to a range of 0..1, with the exception of AoP, which falls within range 0..2π. Therefore, we normalize the AoP by 2π.

By employing this data setup including the scene and camera characteristics, we are now able to characterize the RGB-P camera. In the following, we will present the chosen calculation methods and discuss the achieved results.

## 6. RGB-P Camera Characterization

### 6.1. Model and Architecture of Neural Network

We define the set of all input features for different combinations of ${\left(s_{\Sigma }\left(w\right),{\tilde{S}}_{R},{\tilde{S}}_{G},{\tilde{S}}_{B}\right)}^{T}$ as V with its related output ${\left(c,S\right)}^{T}$ as set W. The minimization problem to be solved is

(40) $$\min_{M_{Pc}}\left\|W-M_{Pc}V\right\|.$$

**Figure 17:**
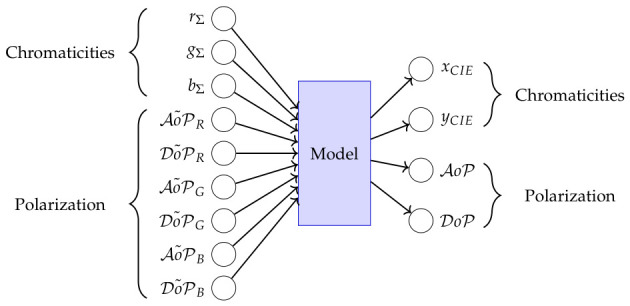
Neural network model transforms camera chromaticities and error-prone polarization features to colorimetric chromaticities, and angle and degree of polarization.

To find a solution, we did look for a base line model as a function approximator, to predict the chromaticity and polarization features (Figure 17).

Models for camera color characterization employing neural networks can be found in [37,38,39,40]. In particular, they predict CIE-XYZ and optimize regarding perceptual colorimetric error distance metrics, such as $\Delta E_{76}$ and $\Delta E_{00}$. In contrast, our model only considers the polarization features and exposure-invariant chromaticity, optimizing based on the mean squared error. Based upon [37] (Cheun02) and [39], we employed an empirical grid search regarding the hyperparameter set shown in Table 2.

Furthermore, we did a k-fold cross-validation of our data by splitting into ten subsets regarding our chosen natural and synthetic reflectance sets and standard illuminant simulators, repeating this process three times. Finally, we employed three models, shown in Table 3. We took two models out of our hyperparameter optimization: RGB-P Char 1, and RGB-P Char 2. But Cheun02 was also adapted to our feature set. Therefore, we modified Cheun02 to include the chromaticity and polarization features in the input and output layer. They used a single gradient descent as the optimizer, as the Adam method was not available at that time. In contrast, our models employ the Adam [41] optimizer. The RGB-P Char 2 architecture incorporates an additional hidden layer, as discussed for RGB sensors in [39].

### 6.2. Training, Validation and Test Set Splitting of Reflectance Sets

**Figure 18:**
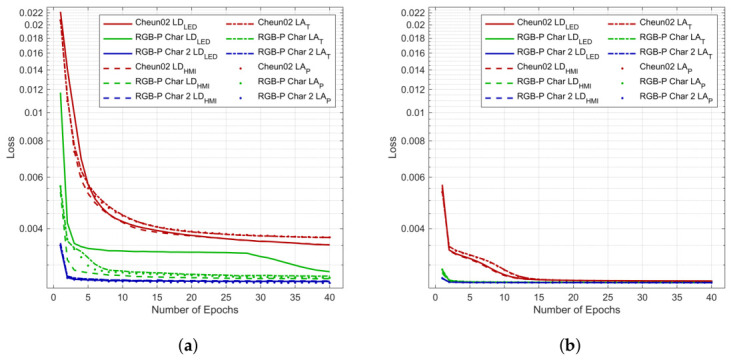
Training Loss: (**a**) $RSC_{1}$. (**b**) $RSC_{2}$.

Table 4 shows the reflectance sets used for training, validation, and testing. For training sets, we have selected natural reflectance sets and synthetic LOGV. The validation set, applied during training, is the CC-DSG test chart reflectance set. The test set is the CC reflectance set expanded by the SOCS-S reflectances, since skin tone appearance is important in visual presentation. The training loss is presented in Figure 18. The trained model sets can be found in [27] (Folder “04RGBPCharacterizationModels”).

### 6.3. Results and Evaluation of RGB-P Camera Characterization

Table 5 and Table 6 display the evaluation results of trained model prediction regarding the test set and illuminations. They show the median and standard deviation for the chromaticity error Δc, as well as for the polarization features errors, ΔAoP and ΔDoP. For training set $RSC_{1}$ with natural reflectances, the chromaticity errors for our methods are comparable and up to three times more accurate than the adapted Cheun02. Having an additional hidden layer in our second model improves the estimation of polarization features. In general, the training using synthetic LOGVINENKO spectra $RSC_{2}$ yields errors that are comparable to those found for the application of natural reflectance sets.

It must be noted that a final implementation of such a model for machine vision or photographic devices needs additional performance evaluation and feasibility studies. One has to consider FPGA, CPU and GPU resource usage, processing time and provided neural network implementations. Here, we have to distinguish between machine vision, and photography and movie applications. If one implements the model in the firmware, using an FPGA, then prediction would be faster. But storage for model parameters is restricted, and neural network implementations are limited. For now, our model parameter set uses a small amount of memory for network parameters. Transferring the model to an FPGA might be feasible, but the calculation performance and power consumption must be evaluated. Since power consumption is a critical factor, especially for mobile devices, one might opt for a model with lower performance, using less resources in the application, such as a model like Cheun02. If the computation is performed in the CPU or GPU domain, storage and neural network implementation are not significant issues. However, copying image buffers between the CPU and GPU domains becomes the bottleneck. This is more important for movie post-production and photography, where processing pipelines consist of arbitrary processing modules. An example for such a pipeline is the creation of a preview and filtering to select the best combination of all polarization channels, regarding visual effects. For now, we have not investigated such implementation aspects. Consequently, further evaluation is required for a given application, addressing these issues.

## 7. Conclusions

We presented a camera characterization method for RGB-P sensor-based imaging devices. It was motivated by observations of different spectral sensitivities for polarization filter pixel elements and chromaticity drift for polarized irradiances. This behavior results in two main issues: first, color accuracy and constancy is not ensured in color signal reconstruction, and second, it leads to inaccurate polarization feature calculations. Additionally, for visualization of RGB-P sensor-based images, one must apply a weighted superposition of colors from the sensor’s polarization channels to render the output.

Our camera characterization method requires the spectral responsivities for two polarization states of sensor irradiance. Therefore, we employed and extended a measurement method and measured spectral responsivities for unpolarized irradiance and linear polarized irradiance. In addition, we measured and discussed the chromaticity drift for linear polarized irradiances.

Based on these measurements, we presented a neural network-based model. It allows an estimation of chrominance and polarization features, as well as ensuring color constancy in visual representation. The model overcomes the limitations of previous techniques developed for unpolarized scene conditions and RGB sensors. We applied our obtained spectral responsivities, and different types of illuminations and reflectance sets to the model for training and evaluation, which yielded robust results. In particular, the use of natural and artificial reflectance sets shows comparable results. The method can be employed for applications in machine and computer vision as well as photography and visual presentation.

However, there is still future work that can be done in our characterization method. Besides the investigated scene reflectance and polarization model, superpositions of diffuse and specular surface reflectances should be investigated. And, among other things, reflectances might be considered, where the spectral reflectance distribution varies over the angle.

We expect trichromatic RGB-P cameras for photography and movie production in the near future. Then, one can consider using our camera characterization framework in this area as well.

## Figures and Tables

**Table 1:** Chosen combinations of illuminants.

	Illuminants	Input	Output
Name			
$LD_{LED}$		ARRI L5 D65	D65
$LD_{HMI}$		Bron Kobold Dlf 575	D55
$LA_{P}$		CMT Kinoflo KF32	CCT = 3200 K
$LA_{T}$		Dedolight Aspheric 2	CCT = 3400 K

**Table 2:** Grid search procedure parameter.

Parameter	Test Cases
Activation Function	Rectified Linear Unit, Linear
Optimizer	Adam, Stochastic Gradient Descent
Number of Hidden Layers	1, 2, 3
Number of Nodes in Hidden Layers	18, 36, 50, 75, 100, 125
Number of Training Epochs	20, 30, 40, 60, 80

**Table 3:** Neural network model parameters.

Name	Layer Structure	Optimization Method	Loss Function Error Metric	Activation Function	Initial Guess Value
**Cheun02**	9/18/4	Stochastic gradient descent	Mean squared error	Rectified linear unit	Random
**RGB-P Char 1**	9/18/4	Adam stochastic gradient descent	Mean squared error	Rectified linear unit	Random
**RGB-P Char 2**	9/100/100/4	Adam stochastic gradient descent	Mean squared error	Rectified linear unit	Random

**Table 4:** Chosen combinations of reflectance sets.

	Sets	Training	Validation	Test
Name				
$RSC_{1}$		ACES-RS	CC-DSG-RS	CC-RS and SOCS-S-RS
$RSC_{2}$		LOGV-RS	CC-DSG-RS	CC-RS and SOCS-S-RS

**Table 5:** Chromaticity and polarization feature error evaluation for daylight illumination; median (*med*), standard deviation (σ).

Model	Data Sets	med(Δc)	σ(Δc)	med(ΔAoP)	med(ΔDoP)	σ(ΔAoP)	σ(ΔDoP)
Cheun02	$LD_{LED};RSC_{1}$	0.012	0.000	0.048	0.006	0.007	0.000
RGB-P Char 1	$LD_{LED};RSC_{1}$	0.010	$6.162\times 10^{-5}$	0.009	0.006	0.008	$6.237\times 10^{-5}$
RGB-P Char 2	$LD_{LED};RSC_{1}$	0.004	$5.306\times 10^{-6}$	0.002	0.001	0.009	$7.337\times 10^{-7}$
Cheun02	$LD_{LED};RSC_{2}$	0.011	0.001	0.006	0.004	0.008	$7.659\times 10^{-5}$
RGB-P Char 1	$LD_{LED};RSC_{2}$	0.005	$8.139\times 10^{-5}$	0.003	0.003	0.009	$6.632\times 10^{-6}$
RGB-P Char 2	$LD_{LED};RSC_{2}$	0.005	$2.614\times 10^{-5}$	0.003	0.001	0.009	$9.839\times 10^{-6}$
Cheun02	$LD_{HMI};RSC_{1}$	0.012	0.000	0.049	0.008	0.007	$6.056\times 10^{-6}$
RGB-P Char 1	$LD_{HMI};RSC_{1}$	0.011	$4.600\times 10^{-5}$	0.001	0.001	0.009	$3.152\times 10^{-6}$
RGB-P Char 2	$LD_{HMI};RSC_{1}$	0.005	$1.458\times 10^{-5}$	0.003	0.001	0.009	$9.417\times 10^{-7}$
Cheun02	$LD_{HMI};RSC_{2}$	0.013	0.001	0.011	0.005	0.008	$8.416\times 10^{-5}$
RGB-P Char 1	$LD_{HMI};RSC_{2}$	0.011	0.000	0.004	0.002	0.008	$6.303\times 10^{-6}$
RGB-P Char 2	$LD_{HMI};RSC_{2}$	0.012	$9.198\times 10^{-5}$	0.004	0.002	0.008	$4.421\times 10^{-6}$

**Table 6:** Chromaticity and polarization feature error evaluation for Tungsten illumination; median (*med*), standard deviation (σ).

Model	Data Sets	med(Δc)	σ(Δc)	med(ΔAoP)	med(ΔDoP)	σ(ΔAoP)	σ(ΔDoP)
Cheun02	$LA_{p};RSC_{1}$	0.009	0.000	0.055	0.007	0.007	0.000
RGB-P Char 1	$LA_{p};RSC_{1}$	0.007	$7.099\times 10^{-5}$	0.005	0.002	0.008	$4.347\times 10^{-6}$
RGB-P Char 2	$LA_{p};RSC_{1}$	0.006	$1.070\times 10^{-5}$	0.002	0.001	0.008	$1.867\times 10^{-6}$
Cheun02	$LA_{p};RSC_{2}$	0.009	0.001	0.007	0.004	0.008	$8.930\times 10^{-5}$
RGB-P Char 1	$LA_{p};RSC_{2}$	0.007	$9.975\times 10^{-5}$	0.003	0.002	0.008	$3.112\times 10^{-5}$
RGB-P Char 2	$LA_{p};RSC_{2}$	0.005	$4.609\times 10^{-5}$	0.002	0.001	0.009	$1.641\times 10^{-5}$
Cheun02	$LA_{T};RSC_{1}$	0.009	0.000	0.059	0.004	0.007	0.000
RGB-P Char 1	$LA_{T};RSC_{1}$	0.005	$4.280\times 10^{-5}$	0.004	0.003	0.008	$1.273\times 10^{-5}$
RGB-P Char 2	$LA_{T};RSC_{1}$	0.005	$9.046\times 10^{-6}$	0.001	0.001	0.009	$4.546\times 10^{-6}$
Cheun02	$LA_{T};RSC_{2}$	0.006	0.001	0.008	0.004	0.008	$5.120\times 10^{-5}$
RGB-P Char 1	$LA_{T};RSC_{2}$	0.004	$5.369\times 10^{-5}$	0.003	0.002	0.008	$1.128\times 10^{-5}$
RGB-P Char 2	$LA_{T};RSC_{2}$	0.004	$2.375\times 10^{-5}$	0.003	0.001	0.008	$4.862\times 10^{-6}$

## Data Availability

The camera and model data sets are openly available at https://hdm-stuttgart.de/open-film-tools/english/camera_characterization/RGBPolChar.zip (accessed on 19 April 2026) [27]. It contains original measured data (01CameraMeasurements) with spectral responsivities for unpolarized irradiation (ResponsivitiesForUnpolarisedConditions) and captured test chart data for polarized irradiation (TestchartImagesForPolarisedConditions), the estimated spectral responses for polarized irradiation (02EstimatedSpectralResponsivitiesForPolarisedConditions), and the camera characterization models (04RGBPCharacterizationModels). Restrictions apply to the availability of SPD of lighting data. Data were obtained from [29] and are available at https://www.hdm-stuttgart.de/open-film-tools/english/cinelight_spectra/OFTP_full-sample-package_v2.zip (accessed on 1 June 2025) with the permission of the authors. Restrictions apply to the availability of skin tone reflectance set data. Data were obtained from [32]. Restrictions apply to the availability of ACES reflectance set data. Data were furthermore obtained from [31] and are available https://github.com/AcademySoftwareFoundation/rawtoaces-data/blob/main/data/training/training_spectral.json (accessed on 1 June 2025) with the permission of Academy of Motion Picture Art and Sciences. Restrictions apply to the availability of ColorChecker Digital SG reflectance set data. Data were also obtained from [42] and are available https://babelcolor.com/index_htm_files/Digital ColorChecker SG.txt (accessed on 1 June 2025) with the permission of the authors. The ColorChecker data applied in this study are available at https://hdm-stuttgart.de/open-film-tools/english/camera_characterization/RGBPolChar.zip (accessed on 19 April 2026) (03ReflectanceSets). These data were derived from the following resources available in the public domain at [43].

## Abbreviations

ACES	Academy of Motion Picture Art and Sciences (AMPAS) Color Encoding System
AMPAS	Academy of Motion Picture Art and Sciences
AoP	angle of polarization
BRDF	bidirectional reflectance distribution function
CC	ColorChecker test chart
CC-DSG	ColorChecker Digital SG test chart
CCT	correlated color temperature
CIE	Commission Internationale de l’Éclairage
DoP	degree of polarization
FWHM	full-width half maximum
HMI	hydrargyrum medium-arc iodide
LED	light-emitting diode
LOGV	LOGVINENKO reflectances [33]
OFTEx	Open Film Tools camera response measurement method [11]
RGB	trichromatic RGB color filter array
RGB-P	trichromatic RGB color filter array and polarization layer
SOCS	ISO/TR 16066 standard
SOCS-S	SOCS skin reflectances
SPD	spectral power distribution

## References

[1] Brewster D. (1815). III. Experiments on the depolarisation of light as exhibited by various mineral, animal, and vegetable bodies, with a reference of the phenomena to the general principles of polarisation. By David Brewster, LL. D. F. R. S. Edin and F. S. A. Edin. In a letter addressed to the Right Hon. Sir Joseph Banks, Bart. K. B. P. R. S. Philos. Trans. R. Soc. Lond..

[2] Atkinson G.A., Hancock E.R. (2008). Two-dimensional BRDF estimation from polarisation. Comput. Vis. Image Underst..

[3] Renhorn I.G., Hallberg T., Bergström D., Boreman G.D. (2011). Four-parameter model for polarization-resolved rough-surface BRDF. Opt. Express.

[4] Kondo Y., Ono T., Sun L., Hirasawa Y., Murayama J. (2020). Accurate polarimetric BRDF for real polarization scene rendering. Proceedings of the Computer Vision–ECCV 2020: 16th European Conference, Glasgow, UK, 23–28 August 2020.

[5] Qiu S., Fu Q., Wang C., Heidrich W. Polarization demosaicking for monochrome and color polarization focal plane arrays. Proceedings of the 24th International Symposium on Vision, Modeling and Visualization (VMV 2019).

[6] Kurita T., Kondo Y., Sun L., Moriuchi Y. Simultaneous acquisition of high quality rgb image and polarization information using a sparse polarization sensor. Proceedings of the IEEE/CVF Winter Conference on Applications of Computer Vision.

[7] Trushkina A., Ryzhova V., Korotaev V. (2016). Calculation of polarization sensitivity of image sensors. J. Phys. Conf. Ser..

[8] Haila T.A., Tausch R., Ritz M., Santos P., Fellner D. (2022). Effect of polarization on rgb imaging and color accuracy/fidelity. Proceedings of the Color and Imaging Conference, Scottsdale, AZ, USA, 6–10 November 2022.

[9] European Machine Vision Association (2021). EMVA Standard 1288: Standard for Measurement and Presentation of Specifications for Machine Vision Sensors and Cameras.

[10] (2012). Recommended Procedures for the Creation and Use of Digital Camera System Input Device Transforms (IDTs).

[11] Karge A., Rieger I., Eberhardt B., Schilling A. (2018). Using Chromaticity Error Minimisation for Fast Camera Spectral Responsivity Measurement. Proceedings of the Color and Imaging Conference, Vancouver, Canada, 12–16 November 2018.

[12] Maxwell J.C. (1865). VIII. A dynamical theory of the electromagnetic field. Philos. Trans. R. Soc. Lond..

[13] Malus E. (1809). Sur une propriété de la lumière réfléchie. Mém. Phys. Chim. Soc. d’Arcueil.

[14] Dirix Y., Tervoort T., Bastiaansen C. (1995). Optical properties of oriented polymer/dye polarizers. Macromolecules.

[15] Guo J., Brady D. (2000). Fabrication of thin-film micropolarizer arrays for visible imaging polarimetry. Appl. Opt..

[16] Von Luther R. (1927). Aus dem Gebiet der Farbreizmetrik. Z. Für Tech. Phys..

[17] Hubel P.M., Holm J., Finlayson G.D., Drew M.S. (1997). Matrix Calculations for Digital Photography. Proceedings of the 5th IS&T Color Imaging Conference, Scottsdale, AZ, USA, 18–21 November 1997.

[18] Hong G., Luo M.R., Rhodes P.A. (2001). A Study of Digital Camera Colorimetric Characterisation based on Polynomial Modelling. Color Res. Appl..

[19] Finlayson G.D., Mackiewicz M., Hurlbert A. (2011). Root-Polynomial Colour Correction. Proceedings of the 19th IS&T Color Imaging Conference, San Jose, CA, USA, 7–10 November 2011.

[20] Finlayson G.D., Drew M.S. (1997). White-Point Preserving Color Correction. Proceedings of the 5th IS&T Color Imaging Conference, San Jose, CA, USA, 7–10 November 1997.

[21] McElvain J.S., Gish W. Cinematic Camera Emulation using Two-Dimensional Color Transforms. Proceedings of the Digital Photography.

[22] Hardeberg J.Y., Schmitt F., Brettel H. (2005). Colorimetric characterization of digital cameras preserving hue planes. Proceedings of the Color and Imaging Conference, Scottsdale, AZ, USA, 8–11 November 2005.

[23] Mackiewicz M., Andersen C.F., Finlayson G.D. (2015). Hue Plane Preserving Colour Correction using Constrained Least Squares Regression. Proceedings of the 23rd IS&T Color Imaging Conference, Darmstadt, Germany, 8–12 November 2015.

[24] Mackiewicz M., Andersen C.F., Finlayson G.D. (2016). Method for hue plane preserving color correction. J. Opt. Soc. Am. A.

[25] Haidinger W. (1844). Ueber das directe Erkennen des polarisirten Lichts und der Lage der Polarisationsebene. Ann. Der Phys..

[26] Solomatov G., Akkaynak D. (2023). Spectral sensitivity estimation without a camera. arXiv.

[27] Karge A. (2026). Data Sets for RGB-P Camera Characterisation. https://hdm-stuttgart.de/open-film-tools/english/camera_characterization/RGBPolChar.zip.

[28] He G.S. (2014). Nonlinear Optics and Photonics.

[29] Karge A., Fröhlich J., Eberhardt B. (2015). A Spectral Database of Commonly Used Cine Lighting. Proceedings of the Color and Imaging Conference, Darmstadt, Germany, 8–12 November 2015.

[30] Karge A. (2015). Data Sets for Commonly Used Cine Lighting. https://www.hdm-stuttgart.de/open-film-tools/english/cinelight_spectra/OFTP_full-sample-package_v2.zip.

[31] Academy of Motion Picture Arts and Sciences Spectral Reflectance Set for Raw to ACES. https://github.com/AcademySoftwareFoundation/rawtoaces-data/blob/main/data/training/training_spectral.json.

[32] (2003). Graphic Technology Standard Object Colour Spectra Database for Colour Reproduction Evaluation.

[33] Logvinenko A.D. (2013). Object-colour manifold. Int. J. Comput. Vis..

[34] Mirzaei H., Funt B. (2014). Gaussian illuminants and reflectances for colour signal prediction. Proceedings of the CIC’22 Color Imaging Conference, Boston, MA, USA, 3–7 November 2014.

[35] Wang Z., Zhao B., Li J., Luo M.R., Pointer M.R., Melgosa M., Li C. (2017). Interpolation, extrapolation, and truncation in computations of CIE tristimulusvalues. Color Res. Appl..

[36] (2005). Recommended Practice for Tabulating Spectral Data for Use in Color Computations.

[37] Cheung T.L.V., Westland S. (2002). Color camera characterisation using artificial neural networks. Proceedings of the Color and Imaging Conference, Scottsdale, AZ, USA, 12–15 November 2002.

[38] MacDonald L., Mayer K. (2021). Camera colour correction using neural networks. Proceedings of the London Imaging Meeting, London, UK, 13–15 September 2021.

[39] Li Y., Li Y., Liao N., Li H., Lv N., Wu W. (2023). Colorimetric characterization of the wide-color-gamut camera using the multilayer artificial neural network. J. Opt. Soc. Am. A.

[40] Kucuk A., Finlayson G.D., Mantiuk R., Ashraf M. (2023). Performance Comparison of Classical Methods and Neural Networks for Colour Correction. J. Imaging.

[41] Kingma D.P., Ba J.A. (2014). A method for stochastic optimization. arXiv.

[42] BabelColor (2023). Spectral Reflectance Set for ColorChecker Digital SG. https://babelcolor.com/index_htm_files/Digital%20ColorChecker%20SG.txt.

[43] BabelColor (2023). Spectral Reflectance Set for ColorChecker. https://babelcolor.com/index_htm_files/ColorChecker_RGB_and_spectra-Avg20_2006.zip.

